# James Patrick Watson MD, FRCP, FRCPsych: Formerly Professor of Psychiatry, King's College London and Honorary Consultant Psychiatrist, South London and Maudsley NHS Trust

**DOI:** 10.1192/pb.bp.116.055368

**Published:** 2017-04

**Authors:** Tom K. J. Craig, Nick Bouras

**Figure:**
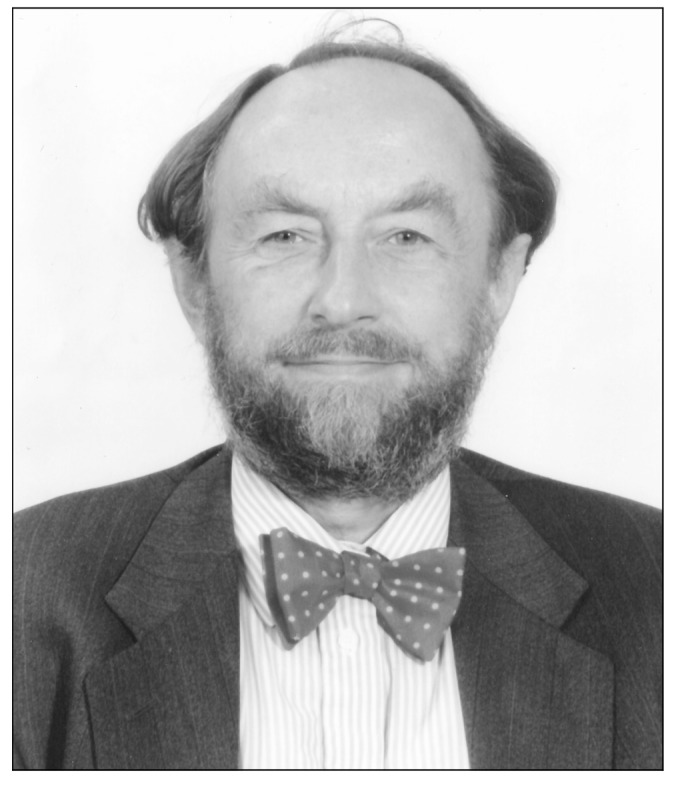


James (Jim) Watson, who died after a stroke on 3 August 2016 aged 80, was among a small band of British psychiatrists who trained in the 1960s and 70s to take psychiatry out of the asylums and establish robust services in general hospital and community settings. They were also responsible for developing a wide range of specialist mental health services. Jim was deeply committed to improving the standards of clinical care, from early implementation of behavioural therapy through in-patient group therapies and the understanding and management of behaviour on hospital wards. He championed the relationship between staff and patient as key to recovery in psychiatry, deploring the move to ever fewer acute beds, reductions in staffing levels and organisational changes that resulted in fractured continuity of care and consequent erosion of the essence of good mental healthcare.

His clinical interest was reflected in his research, which included evaluations of community mental health, telemedicine and treatments for psychosexual disorders, for which he established one of the earliest specialist multidisciplinary clinics and training programmes in Britain. With his personal style, he led a vibrant, outward-facing, creative and very happy department, in which a serious commitment to excellence went along with a refreshing lack of pomposity and a keen sense of work being enjoyable. This was in no small part due to Jim's dedication to improving the quality of psychiatric services, not least by ensuring excellence in the education and training of psychiatrists and by making sure that medical students had a varied and stimulating exposure to psychiatry.

Under his leadership, Guy's Hospital Medical School had the enviable reputation of having the highest proportion of medical students opting for a career in psychiatry. In the postgraduate field, he was an inspirational leader of the South East of England training scheme for psychiatry, chairman of the Royal College of Psychiatrists' Specialist Training Committee and chairman of the Association of University Teachers of Psychiatry. In the mid-1990s he launched an MSc in mental health studies – a programme directed at professionals from all disciplines involved in delivering mental health services. This course was extraordinarily successful: consistently oversubscribed, with unprecedented numbers of applicants. Its success spawned further collaborations with university departments overseas, notably in Egypt and the Middle East, where he worked with colleagues to develop a diploma in psychiatric practice for wider dissemination across the region. His determination to improve mental healthcare led him to a lengthy involvement with mental healthcare in Pakistan. From the early 1990s, he collaborated with colleagues there, visiting regularly and helping to train staff for mental health clinics in rural settings that have now expanded to more than 15 centres, some of which are co-located with a mosque and madrasa. Jim was also connected with several other international projects involving, among other countries, Greece and the former Yugoslavia.

Jim was the eldest of three sons. His father was a teacher and his mother a doctor. He attended the Roan School for Boys in Greenwich, where he excelled academically and in sport. He studied medicine at Trinity College, Cambridge, where he was a senior scholar. In 1957 he transferred to King's College Hospital Medical School for clinical studies, qualifying in April 1960. It was there that he met his fellow student and future wife Christine Colley – they were married in April 1962.

After training in psychiatry at the Bethlem Royal and Maudsley Hospitals and Institute of Psychiatry, he was appointed as consultant and senior lecturer in psychiatry at St George's Hospital London in May 1971. He was appointed to the Chair of Psychiatry at Guy's Hospital Medical School in September 1974, steering his department through the union with St Thomas's Hospital in 1982 and onward to the final merger with King's College in 2000. In addition, he served as honorary consultant psychiatrist to the British Army from 1980 to 2000 and was the vice-president of the Royal College of Psychiatrists from 1998 to his retirement.

After retirement, Jim continued to contribute actively to the field, providing teaching and mentorship to psychiatrists in the Sussex Partnership NHS Foundation Trust. He maintained his collaboration with colleagues in Pakistan, advising on setting up a new School of Nursing as well as the development of a service and training resource for children with learning disabilities and autism. He took part in continuing education meetings with colleagues in Cheltenham, chaired a patient support group in a local general practitioner practice and was trustee of the Soundwell Music Therapy Trust which provides music therapy for people suffering from mental ill health.

Jim enjoyed a very happy family life, having four sons whose diverse careers in music, international school teaching, hospital medicine and clinical psychology were a great source of pride and affection. In 2002, he and Christine exchanged London life for a Cotswold home where he could indulge his enthusiasm for vegetable gardening – a throwback to his maternal ancestors who had traded as market gardeners, supplying mustard and cress to Queen Victoria.

Jim's passing will be felt as a great loss to psychiatry and by the many clinical and academic colleagues who had the privilege of knowing and working with him.

